# Pro-Thrombotic Activity of Blood Platelets in Multiple Sclerosis

**DOI:** 10.3390/cells8020110

**Published:** 2019-02-01

**Authors:** Joanna Saluk-Bijak, Angela Dziedzic, Michal Bijak

**Affiliations:** Department of General Biochemistry, Faculty of Biology and Environmental Protection, University of Lodz, Pomorska 141/143, 90-236 Lodz, Poland; angela.dziedzic@unilodz.eu (A.D.); mbijak@biol.uni.lodz.pl (M.B.)

**Keywords:** blood platelets, multiple sclerosis, pro-thrombotic activity

## Abstract

The available data, including experimental studies, clearly indicate an excessive intravascular activation of circulating platelets in multiple sclerosis (MS) and their hyper-responsiveness to a variety of physiological activators. Platelet activation is manifested as an increased adhesion and aggregation and is accompanied by the formation of pro-thrombotic microparticles. Activated blood platelets also show an expression of specific membrane receptors, synthesis many of biomediators, and generation of reactive oxygen species. Epidemiological studies confirm the high risk of stroke or myocardial infarction in MS that are ischemic incidents, strictly associated with incorrect platelet functions and their over pro-thrombotic activity. Chronic inflammation and high activity of pro-oxidative processes in the course of MS are the main factors identified as the cause of excessive platelet activation. The primary biological function of platelets is to support vascular integrity, but the importance of platelets in inflammatory diseases is also well documented. The pro-thrombotic activity of platelets and their inflammatory properties play a part in the pathophysiology of MS. The analysis of platelet function capability in MS could provide useful information for studying the pathogenesis of this disease. Due to the complexity of pathological processes in MS, medication must be multifaceted and blood platelets can probably be identified as new targets for therapy in the future.

## 1. Introduction

Multiple sclerosis (MS) is a major human putative autoimmune, demyelinating, and degenerative disease of the central nervous system (CNS) with a variety of pathophysiological mechanisms, such as axonal injury, neuronal damage, inflammation, demyelination, astrogliosis, and remyelination [1,2]. Processes above are not evenly represented in the entire MS population, but can selectively prevail in individual patients. Namely, variety present in phenotypic expression of MS does have an essential effect on the response to treatment of MS patients and the course of the disease. However, due to the intricacy of MS, it is heavily to predict the course of the disease in a particular patient. This neurodegenerative disease is characterized by both relapses and insidious progression, and is notably varied in clinical course, severity, and symptomatology [3]. MS as a highly heterogeneous disease is considered to be a conglomerate of neurological syndromes. Clinically, there are four subtypes of MS: relapsing–remitting (RRMS), secondary progressive (SPMS), progressive-relapsing (PRMS), and primary progressive (PPMS). In around 85% of cases, MS begins with an acute neurological episode, which is the first clinical syndrome of RRMS. Over time, as the disease progresses, the fluctuations between periods of neurodegeneration, caused by inflammation, leading to demyelination, and repair mechanism (remission), are observed. About 15–20 years after the first symptoms, about three-quarters of cases of RR transform permanently to SP [4]. This second form is the more debilitating stage of the disease, characterized by a gradual and irreversible neurological decline. Axonal degeneration is the dominant pathogenic factor in the progressive form of MS and correlates with disability, and brain and spinal cord atrophy [5]. In patients, degenerative changes occur in different areas of the brain, spinal cord and peripheral nerves, therefore neurological symptoms are varied. Demyelinating lesions in the spinal cord are a common cause of spastic bladder [6]. Partial interruption of nerve impulses passing along the spinal cord is the cause of weakening the strength of the muscles provided by these nerves, which therefore do not function normally. Patients have symptoms of paresis (most often lower limbs), which means that all or some limbs or parts of the limbs may be paralyzed. The paresis is often accompanied by sensory disturbances, which consists of a simple weakening of the surface sensation or unusual sensations [7].

The pathophysiological mechanisms of this multi-component disease are difficult to clearly characterize. The nature of the pathophysiology of autoimmunity is very complex and involves the various type of cells. The etiology of MS is still unknown, however, accumulating body of evidence points to a complex interplay of genetic and environmental factors disturbing the functioning of the immune response. MS pathomechanism is associated with many factors, the most important of which include immune system dysfunctions combined with biochemical disturbances and disruption of the blood–brain barrier (BBB) [8]. The cause of immune dysregulation in MS seems to come from dysfunctions of dendritic cells namely antigen-presenting cells (APCs) which pass through the BBB and initiate the differentiation of memory T cells into pro-inflammatory T helper lymphocytes (principally, Th1 and Th17). Autoreactive T cells get into the CNS, where the pro-inflammatory cytokines are released from their granules, thereby activating macrophages and microglial cells. Pro-inflammatory mechanisms in microglia can favor the disease’s progression. Induced cells secrete additional pro-inflammatory cytokines as well as generate oxygen and nitric oxide radicals, resulting in inflammation of white matter and consequent axonal loss [9].

The disruption of the permeability, especially for small molecules and blood cells, that enter the bloodstream, contributes to the infiltration of a large number of leukocytes, thus inducing the neurotrophic changes causing demyelination in the CNS. However, there is growing evidence that indicate the co-existence between co-stimulatory pathways and the function performed by inflammatory cells, both in the periphery nervous system (PNS) and in the CNS. Platelet activation significantly affects the interactions between leukocytes and endothelial cells [10]. It is believed that endothelial and platelet dysfunction contribute significantly to the development of neurodegeneration in MS patients. Because activated platelets may adhere to inflamed changed endothelium or proteins present on the subendothelial layer of blood vessel walls, as well as on the tendency to form platelet-leukocyte complexes, allows them to participate in the development of inflammation. Platelets through mechanisms dependent on their receptors, or biologically active compounds secreted from their granules. Additionally, platelets can directly activate leukocytes as well as dendritic cells. The list of well-known agents secreted from platelets granules is wide, containing of broad range of pro- and anti-inflammatory factors that regulate homo- and heterotypic aggregation, angiogenesis, chemotaxis, matrix degradation, and signalling pathways in selected cells [11,12]. Under the neuroinflammatory conditions caused by the disturbance of BBB, blood platelets quickly adhere to changed endothelium at the sites of vascular injury and became activated. Multiple interactions between activated blood platelets and inflammatory cells in the endothelium contribute to neurovascular inflammation. Blood platelets adhering to endothelial cells support the process of diapedesis and leukocyte infiltration into the affected vessel. After activation, platelets secrete large amounts of various mediators and express a number of cell surface receptors which stimulate inflammatory cells. Interactions cell–cell supply crucial mechanisms by which platelets are linking the thrombosis, inflammation, and other related processes [13,14].

## 2. Increased Risk of Cardiovascular Disorders

As already mentioned, MS is a demyelinating and inflammatory disease of CNS, however, it is closely related to the damage of intracerebral blood vessels, mainly as a result of increased BBB permeability, and/or as a consequence of the closure of the blood vessel [14]. Multiple research has examined the risk of vascular disorders in MS patients. The latest reports on vascular disease confirmed an augmented risk of cardiovascular disease, particularly ischemic stroke, thrombosis and myocardial infarction in MS. Aforementioned ischemic events are strictly associated with coagulation cascade as well as abnormal platelet pro-thrombotic activity and function [15,16,17,18,19,20,21,22].

Well-documented epidemiological reports show an augmented risk of cardiovascular disease in patients with MS, and some studies have found that the risk of death due to ischemic stroke or myocardial infarction in MS was significantly greater (over 30%) than for the age-matched overall population. Moreover, according to statistics, the cause of death in more than 10% of MS patients considered a stroke [17,22]. Analysis carried out on more than 6000 deaths of patients catalogued in the Danish National Registry of MS Patients disclosed that death by cardiovascular diseases was the most common cause of death, except for the disease itself [21]. A cohort study of 13,963 Danish patients with MS found an increased risk of ischemic stroke soon after diagnosis of MS (within a year), and showed that the raised risk was most appearing in young adults with MS (≥ 56 years). It is worth emphasizing that there were no differences or heightened risk for hemorrhagic stroke [16]. The Swedish study discovered the opposite correlation between age and risk of stroke in patients with MS. In the first phase of the disease (RRMS), MS relapses are often misdiagnosed as stroke, and this may be the cause of misinterpretation in the Norwegian study showing elevated risks of stroke at the beginning of the disease. Study conducted in Sweden on a group of 8281 patients with MS and the control group confirmed the risk of myocardial infarction, stroke and heart failure. In addition, it has been documented that the risk is clearly higher in women than men. As the authors suggest, the increased risk might result from common pathological factors present in both MS and cardiovascular diseases (such as inflammation, oxidative stress, thrombotic agents) [23]. The nationwide study in Denmark that included MS patients from 1980–2005 confirmed low frequency of cardiovascular diseases preceding MS, but significantly high after the MS diagnosis. This suggests a common etiological mechanism for onset of MS and vascular disorders, or that clinically active MS is a trigger mechanism for the development of cardiovascular diseases [24]. Furthermore, the latest research, inspired by these observations, confirmed that the presence of hypertension and heart disease may contribute to advanced brain atrophy in MS patients over five-year follow-up period. Thus, concurrence of MS and cardiovascular diseases may contribute to additional neurodegenerative injury [25]. Therefore, as the authors of the recent work emphasize, clinicians need to incorporate the prevention and management of co-occurring cardiovascular diseases during treating patients with MS, which requires the establishment of pathogenesis mechanisms. It is important to characterize the causal mechanisms underlying the associations between cardiovascular conditions and adverse MS outcomes. The more so because a number of recent studies seem to confirm this hypothesis that the optimization of cardiac comorbidities, especially hypertension, may result in less severe outcomes for patients with MS [26,27].

Demyelinating lesions within specific areas in the central nervous system may be involved in the pathogenesis of autonomic dysfunction in MS [28]. It has been proven that analyses of heart rate variability (HRV) detect early autonomic involvement related with cardiovascular risk in patients with neurologic diseases [29]. A recent study disclosed that newly diagnosed patients with RR MS had decreased HRV compared to healthy controls, indicating a dysfunction of the autonomic nervous system, which may be a predictor of many cardiovascular [30].

Moreover, MS these patients lead a more sedentary lifestyle, which promotes the occurrence of stroke [23], and above all, is related with the risk of venous thrombosis which seemed to be raised in MS patient in comparison with control group [31,32]. Several extensive studies on venous thromboembolism in MS have shown the association between the coagulation system and inflammation [33,34,35]. Platelets are involved in MS pathogenesis, playing an essential part in the innate immune response, by linking two processes, coagulation, and inflammation [36,37,38]. Thus, activation of platelets can implicitly be a vascular risk factor in MS.

It is well-known that the lack of physical activity is closely related to the emergence of so-called metabolic syndromes—such as obesity, hypertension, dyslipidaemia, elevated glucose concentrations, and higher levels of high-density lipoprotein (HDL) and cholesterol—Which significantly increase the risk of vascular incidents. According to research carried out by Laroni et al. indicate that treatment of MS patients with disease-modifying drugs (DMDs), especially glatiramer acetate, fingolimod, and dimethyl fumarate significantly enhances the risk of cardiovascular diseases [39]. While it appears that MS patients are at greater risk of cardiovascular-related diseases, the fundamental reason is multi-factorial and unclear.

## 3. Pathological Activation of Platelets in MS

Although the contribution of platelets to inflammation is well established and the inflammatory response has been recognized to be critical in neuronal diseases such as MS, the participation of blood platelets in neurodegeneration is weakly investigated till now. In the 1950s, there were only a few early studies on the importance of platelets in the CNS demyelination, which presented elevated platelet adhesion in the initial stage of MS (RRMS) [40]. Since that time, several other papers have been published confirming blood platelet pathologies in patients with MS. However, the role of platelets in inflammation appears to be still comparatively unkempt in neurological studies [36]. Although examination of platelet physiology in MS is infrequent in the current literature, there are some reports, including our study, that clearly demonstrates chronic platelet activation in MS. The vast majority of data on the pathophysiology of platelets in MS is based on research conducted among patients in the first stage of the disease (RRSM). Our previous findings focused on patients in the SP phase of MS, and proved that platelet activation in MS could be an epiphenomenon consequent to the disease’s development, presumably secondarily to endothelial injury, which exposure platelets on variable subendothelial stimuli [41,42,43]. Langer et al. demonstrated that activated blood platelets are stored in chronically activated demyelinating lesions of the inflamed murine brain and spinal cord subjected to experimental animal model of autoimmune encephalomyelitis (EAE). Then, histological analysis confirmed that platelets were abundant in patients with MS [44].

MS affects only human, however, several animal models of this disease have been identified. The most commonly used models include EAE and cuprizone model. The choice of the appropriate model depends on the method of inducing the process of demyelination in the CNS. In the EAE model, induction occurs with myelin antigens or passive transfer of myelin-specific T lymphocytes induces an inflammatory demyelination in the animal CNS. The copper model is based on the toxic induction of demyelination with the participation of copper chelator cuprizone (bis-cyclo-hexanone oxaldihydrazone). There are many of research based on the cuprizone animal model. One of them is research conducted by Berghoff et al., which was based on the impact of demyelination on BBB hyperpermeability. This research indicates that proinflammatory molecules such as interleukins (IL-6 and IL-1β), tumor necrosis factor (TNF), and chemokine CCL2 are the most likely candidates to contribute to BBB disorders in the cuprizone animal model [45]. The studies of Sheremata et al. using a flow cytometer also supported significant platelet activation in RRSM, expressed as a high percentage of microparticle pool and platelet aggregate formation, and increased surface exposure of P-selectin (marker of platelet activation) which plays an imperative part in their crosstalk with the endothelial cells and leukocytes [46]. Consistently with other studies, our research presents a high degree of chronic activation of platelets in the circulatory system of SPMS patients. We clearly indicated that platelets from SPMS are more excitable to agonists, and their response expressed as adhesion and aggregation is notably stronger than blood platelets obtained from the control group [41,43].

Blood platelets are the main cell effector in hemostasis, coagulation, and pathologic thrombosis. Adhesion of blood platelets at sites of vascular injury is a crucial first step in hemostasis and thrombosis, prompted by the exposure of subendothelial matrix compounds, mainly collagen or other thrombogenic proteins. These receptor-mediated processes involve adhesion surface molecules and lead to signal transduction, platelet aggregation, degranulation, and secretion of pro-thrombotic factors from platelet granules. Further reinforcement of platelet activation and secondary acquisition of extra blood platelets contributes to the creation of the haemostatic plug. Various physiological stimuli activate signalling pathways, consequently causing platelet adhesion, degranulation (secretion of compounds magazine in their granules), excretion of microparticles, and inducing expression of the receptors that finally mediate platelet aggregation. Blood platelets excessively activated by endogenous agonists contribute to various types of pathogenesis [47]. Platelets are the main elements of cellular hemostasis, but likewise play a significant role in the coagulation cascade. The presence of activated coagulation factors to facilitate the coagulation of plasma and the final conversion of fibrinogen is necessary. Platelets are actively involved in the coagulation cascade by providing a phospholipid surface to amplify of local generation of thrombin, and by releasing the stored coagulation factors. Activated platelets participate in clot propagation and stabilization. Furthermore, they contribute to remodelling and clot retraction [48]. 

**Figure 1:**
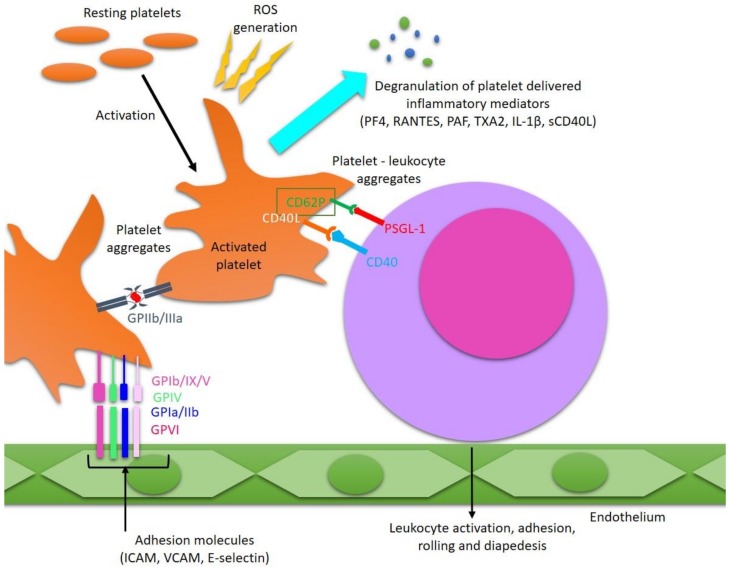
Pathomechanism of platelet participation in MS. Platelets during activation can interact with leukocytes, endothelial cells, and other platelets creating cell complexes by adhesion molecules and glycoprotein membrane receptors, such as GPIb-IX-V complex, GPIV, GPVI, GPIa/IIb, GPIIb/IIIa, CD62P, and CD40L. Stimulated platelets promote activation, adhesion and enhance transmigration (diapedesis) leukocyte by endothelium in response to inflammation. GPIb-IX-V, GPIa/IIb, GPIIb/IIIa, GPIV, and GPVI—Platelet transmembrane glycoproteins; P-selectin—Granular membrane protein of platelets and a cellular adhesion molecule; E-selectin—Cell adhesion molecule, expressed only on activated endothelial cells; CD40L—Protein, primarily expressed on activated T cells and blood platelets; CD40—Costimulatory protein, on antigen presenting cells; PSGL-1—P-selectin glycoprotein ligand-1; ICAM—Intercellular adhesion molecule; VCAM—Vascular cell adhesion molecule.

Platelet quantity and activation status are critical to physiological and pathological hemostasis. Understanding the molecular mechanisms responsible for the increased platelet activation in MS can have important implications for finding effective therapies. A common feature of myocardial infarction and stroke is vessel occlusion. Pathological activation of blood platelets and formation of thrombotic lesions within the coronary arteries are considered to be the major cause of myocardial infarction [49]. It has been proven that over-activation of platelets plays a crucial role in thrombotic closure of the coronary vessels, causing the reduction or cessation of flow in the coronary arteries. A characteristic feature of patients with acute myocardial infarction is chronic platelet activation and enlarged aggregation within the coronary circulation. A similar pathophysiology is characteristic of ischemic stroke, which is a consequence of cerebral vascular occlusion [50]. The increased clotting cascade generates excessive amounts of thrombin—The main protease of coagulation, which converts soluble plasma protein, fibrinogen, into insoluble fibrin. Fibrinogen is a key protein of the clotting process, which transforms into an insoluble clot and is responsible for venous thrombosis due to blocking of blood vessels. An epidemiological study reported that the duration of MS is one of the primary risk factor of stroke and deep vein thrombosis [17]. The development of coagulation cascade correlates with platelet activation. The generation of the great amounts of thrombin responsible for platelet activation is caused by activation of coagulation factors, which are also involved in the development of pro-inflammatory response in patients with MS. Performed proteomic analysis of active inflammatory changes present in demyelination plaque in MS confirmed the participation of coagulation cascade factors [51]. After activation, platelets synthesize a broad array of proteins [52]. Blood platelets also exhibit features of inflammatory cells. Platelets, despite the absence of a nucleus, are able to synthesizing proteins by transcripts passed from megakaryocytes [53]. In recent years, our knowledge of the pool of bioactive agents in platelets has continued to grow, due to the introduction of new technologies for investigating the molecular mechanisms of platelet prothrombotic activity. Studies of the genetic basis of prothrombotic and pro-inflammatory platelet activity in MS include analysis of proteomics, lipidomics, and mRNA transcripts. As shown, tranquil platelets synthesize a lot of proteins including fibrinogen thrombosthenin, membrane glycoproteins (GPIb, IIb, and IIIa), thrombospondin, and von Willebrand factor. Moreover, platelets after activation use an additional synthetic pathways to manufacture novel proteins, which regulate inflammation [52]. The platelet pro-inflammatory and pro-thrombotic activities significantly contribute to the development of many currently occurring diseases. Platelet glycoprotein receptors mediate platelet accumulation by platelet-vessel wall interactions, as well as leukocyte recruitment (platelet-dependent). As platelets participate in the development of an inflammatory response, their integrin receptors have a high proportion of interactions with leukocytes and in the mediation of immediate adhesion of platelet to the endothelium layer or to the subendothelial extracellular matrix, which is exposed at the sites of the blood vessel injury. Various platelet adhesion receptors have been involved in these processes, such as the integrin GPIIb/IIIa, glycoprotein (GP) VI, or the GPIb/IX/V complex. The microarray analysis of human MS plaques has shown an augmented expression of transcripts for chief platelet integrin GP IIb/IIIa—the fibrinogen receptor, which is critical to platelet aggregation. Findings derived from chronic lesions of MS patients were confirmed by studies on activated platelets present in the murine brain in the course of EAE. It has been proven that after inhibition of GP IIb/IIIa, the paralysis and experimental animal model of EAE were respectively ameliorated and reduced [54,55]. Pathomechanism of platelet participation in MS is presented on Figure 1.

## 4. Platelets’ Involvement in the Development of Inflammatory Reactions in MS

Except for the pivotal role in primary hemostasis, platelets are essential for the maintenance of normal function of endothelial barrier. One of the cardinal symptoms of inflammation is the increased vascular permeability caused by changes in the functioning of the barrier [44]. It was observed that depletion of platelet counts markedly alleviated the symptoms and inhibited the inflammation process. Decreased number of platelets resulted in a diminished inflammatory response in EAE murine model, including weaker diapedesis of leukocytes into the inflamed spinal cord and limited production of inflammatory molecules, like cytokines and chemokines. Reduction in the number of damaged axons and plaque of demyelination were manifested as silencing symptoms in the animal EAE model. Platelet depletion at the inflammatory phase of EAE in mice did not influence the immunization phase, however, resulted in significant reduction of disease development and progression. The mentioned data suggested that platelets play an essential role in the inflammatory response in the course of MS [44,54]. It is now obvious that activated platelets control local inflammatory responses through the interplay between endothelial cells and infiltrating leukocytes through immunomodulatory ligands derived from platelets. The important role of activated platelets in the development of inflammation is the result of their adhesive inclination toward inflamed endothelium and protein components located in the sub-endothelial layer of blood vessel walls, as well as being an effect of platelet-leukocyte complex formation. It is assumed that the interplay between blood platelets, leukocytes and endothelium is a direct cause of the disruption of BBB. Because of this, it is considered the first critical step in the initiation and development of subsequent stages of the pathogenesis of MS, causing infiltration of lymphocytes, and then to the creation of demyelinating lesions in CNS [56]. Activated platelets release leukocyte chemoattractants and express abundant molecules present on the cell surface, like intercellular adhesion molecule (ICAM)-2, CD62P, and a broad range of glycoproteins, which play a part in intracellular interactions, and are significant in the stabilization of interactions between leukocytes and vasculature. According to documented reports, platelets attach to T lymphocytes via a CD11a/ICAM-2 interaction [57], while neutrophils, monocytes, and dendritic cells via CD62P/CD162 (P-selectin glycoprotein ligand-1; PSGL-1) interaction [58,59]. Platelet adhesion to inflamed endothelium leads to CD62P expression by platelets, thus mediating leukocyte co-localization at sites of BBB injury [60]. Monocytes are the major leukocytes that are the most often complexed with platelets, in vivo. Activated blood platelets are well-known to release a lot of soluble form of CD62P (sCD62P), which is responsible for inducing monocyte differentiation into dendritic cells, and polymorphonuclear cell activation. Taking into consideration that occurrence of various subpopulations of monocytes in the circulatory system is varies substantially and is correlated with differing disease states. Wherefore, interactions of blood platelets may have clinical implications connected with elevated numbers of certain monocyte subpopulations [61,62]. 

Several mentioned studies have shown that activated platelets can modulate dendritic cell maturation and their activation. Dendritic cells represent highly specialized APCs and presence both in the innate and adaptive immune system by stimulating naive, memory, and effector T-cells, promoting B cell activation, and interacting with NK cells [63]. There are more evidences concern the enhanced number of dendritic cells in atherosclerotic plaques, and their interaction with blood platelets [64]. Thus, platelets are active components of the immune system, due to mediation of dendritic cells’ ability. In addition to their hemostatic role, blood platelets have been demonstrated a crucial role in both innate and adaptive immune system [9]. Elzey et al. confirmed that the expression of CD40L on platelets was desired to induce dendritic cell maturation, switch into B cell isotype, and enhance CD8+ T cell responses in a murine model, in vivo [65]. Platelets are the major source of membrane glycoprotein of the TNF family—CD40L—Which was primarily identified on activated CD4+ T cells. The interaction between CD40L present on T cells with CD40 on B cells is of supreme significance for the development and functioning of the humoral immune system. The CD40/CD40L interaction is crucial in the production of B cell antibody dependent on the thymus. However, CD40 is present not only on B cells, but it is also found on endothelial cells, monocytes and macrophages, which indicate that CD40/CD40L pathway has multiple functions, in vivo. Expression of CD40L on the platelet surface occurs within a few seconds. CD40L present on activated platelets induces endothelial cells to secrete pro-inflammatory molecules, including chemokines and expression of adhesion molecules. The CD40/CD40L pathway is designed to induce signals for the gathering of leukocytes at the site of developing inflammation [66,67,68]. The platelet signalling pathway CD40/CD40L is a crucial player in many various autoimmune diseases, including MS, and is regarded as a marker of the early phase of autoimmune inflammation. Over 95% of the soluble form of CD40L (sCD40L) in plasma comes from the granularity of activated platelets. Burman et al. demonstrated an elevated level of sCD40L in the cerebrospinal fluid from RRMS patients. The authors also suggested that sCD40L from activated blood platelets can disturb BBB permeability in the CNS [69].

It is well-known that blood platelets are a rich source of biologically active molecules, like inflammatory mediators. In the circulatory system, platelets after activation release various types of components that are stored in the highly organized characteristic cell granules. A plurality of biologically active compounds released from platelet granules, sometimes at high concentrations, can disturb the permeability of BBB. Due to activation of T-lymphocytes which are responsible for the emergence of new inflammatory injuries in MS [70]. It has been shown a raised quantity of platelet granule secreted markers, such as a β-thromboglobulin (β-TG) and platelet factor 4 (PF4). Furthermore, there is a positive correlation between PF4 plasma level and the severity of the disease [71]. During activation platelets release a lot of potent inflammatory signalling molecules, such as cytokines and chemokines, which may be involved together with matrix metalloproteinases (MMPs) in developing of inflammation [72]. PF4 also known as CXC chemokine ligand 4 (CXCL4) and platelet chemokine ligand 5 (CCL5) also known as RANTES (regulated on activation, normal T cell expressed and secreted) form heterodimers, leading to promote monocyte recruitment to the endothelium. Moreover, PF4 supports neutrophil degranulation and their adhesion to endothelial cells, promotes monocytic differentiation into macrophages, and causing phagocytosis and generation of free radicals [73].

Platelets after activation produced inflammatory lipid mediators such as eicosanoids, like thromboxane A2 (TXA2), or platelet activating factor (PAF) which transmit outside‑in signals to diverse cell types involved in innate immune defence, furthermore, PAF likewise induces severe endothelial barrier leakiness. PAF is endogenous phospholipid of proinflammatory, hemostatic, and vasoactive properties, is naturally synthesized by a various of cell types, such as platelets, neutrophils, monocytes, endothelial cells, and neurons. This metabolite of arachidonic acid induces, both platelet and neutrophil adhesion and activation. Additionally, provide variations in local cerebral blood flow and blood rheology. PAF as a solid platelet activator, causes platelet synthesis of IL‑1β—The pro-inflammatory cytokine which has been suggested to have a major role in vascular inflammation by activation of the cytokine regulatory network [74]. PAF secreted on account of cooperation between blood platelets and leukocytes would facilitate the permeability of the BBB and all the more so as one of the most distinguished effects of PAF is disruption of junctions in the endothelial layer [75].

Similarly, platelet-derived microparticles (PMPs), which contain a large amount of the platelet-derived membrane proteins, such as the MMPs that have been discerned as a key player in disruption of BBB in patients with MS [13]. Microparticles derived from different types of cells are follicular structures mostly produced throughout activation and cell death. It has been observed that they contain a variety of molecules with agonist and antagonist activities inside of their structures and on their surface. Therefore, it has been suggested that microparticles might facilitate pathological effects in several autoimmune diseases and actively contribute to the chronic inflammatory process [76,77]. It has also been proposed that excessive production of microparticles as a result of chronic cell activation can predispose to autoimmune diseases [78]. Most of blood cells, released microparticles, but PMPs constitute 90% all of circulating cell particles, and it is well documented that their increased level is typical of some diseases. In recent years, the number of studies that confirmed a significant contribution of PMP in such autoimmune diseases as systemic lupus erythematosus (SLE) and rheumatoid arthritis (RA), has grown. Reports indicate not only augmented levels of PMPs in RA and SLE, but likewise indicate their correlation with disease activity [79,80,81].

Interaction between platelets and leukocytes is thought to signify an important trigger for induction of proteolytic MMP activity. Platelet-derived MMPs fundamentally contribute to a variability of pathologies by supporting cell migration, tissue degradation, and finally inflammation. Platelets and exude a diversity of MMPs, such as MMP-1, MMP-2, MMP-3, MMP-9, and MMP-14. Moreover, platelets elevate MMPs concentrations at sites of vascular detriment by leukocyte recruitment on the endothelium cell surface and stimulation both of them to produce the MMPs [81,82]. Platelet-monocyte interactions induce extracellular matrix metalloproteinase inducer (EMMPRIN)-dependent synthesis and release of NF-κB-mediated MMP-9, MMP-14, and inflammatory cytokines, like IL-6, IL-8, and TNF-α in monocytes. EMMPRIN, also known as CD147 belonging to the immunoglobulin-like receptor family is stored in the alpha granules of platelets and after agonist stimulation moves into the surface of platelet membrane [83]. The interaction between neutrophils and platelets mediated by neutrophilic P-selectin glycoprotein ligand-1 (PSGL-1) and platelet P-selectin (CD62P) stimulate platelets to deliverance of MMP-2, that induce platelet-neutrophil aggregation [84]. Moreover, platelets release a multiplicity of pro-inflammatory chemokines and cytokines, that induce synthesis of MMPs in numerous cell types. One of them is platelet CD40L, which induces MMP-1, MMP-2, MMP-9, and MMP-14 on endothelial cells [85].

Blood platelets probably can also perform a role of direct effector cells during immune processes due to their increased surface expression of IgE receptors, which is an important immune defence mechanism. Binding of IgE by platelet membrane lids to platelet activation results in their aggregation, formation of heterogeneous platelet-lymphocyte conjugates and release of cytotoxic mediators [86,87,88].

As we demonstrated in this paper platelets have a multi-form participation in development of MS. Therefore, we should take into account that platelets may become a target for new drugs administered in the course of MS mainly to relieve symptoms and prevent inflammation. The reduction of their activity will also have a significant effect on the level of risk of selected cardiovascular diseases in MS. All of the studies cited herein highlight the significance of platelet activation in MS. This leads to the supposition that blood platelets are probably constantly activated in development of MS, and are engaged in the pathogenesis of the disease by promoting inflammatory processes. Nevertheless, as emphasized by the authors of most researches, the role of blood platelets in pathogenesis and development of MS stay unknown. Further studies are still needed to validate the correctness of the information received to date, and to more broadly determine the role of platelet activation in the neuro-pathogenesis of MS. All the more so, as the clear mechanisms of increased platelet activation in MS also remain unknown. Further research on platelets in MS are particularly important in clarifying the reason for increased risk of ischemic events observed in MS. Further analysis of platelet function in MS lesions might elucidate new features of pathology, and could open the opportunities for therapy. The precise definition of the mechanisms of increased platelet activity in MS will determine the new therapeutic goals of this disease. The existing reports indicate that blood platelets could be a possible target for pharmacotherapy in MS, then their over-activation can be implicated in the development of the neuroinflammatory process correlated with neurodegenerative diseases. The summarizing table of proven changes in platelet activity and functions in MS has been presented in Table 1.

The different treatment options of MS are based on different mechanisms of action, influencing their use and effect. MS is a disease of unclear etiology, which makes its treatment difficult and is limited only to symptomatic treatment, which slows down the development of the disease. Perhaps in the future, disease-modifying drugs will be direct against platelet activation and especially to combat their interaction with immune cells for prevent the progression of inflammatory process. Currently, several disease-modifying drug therapies are available for the treatment of MS and are the standard treatment for MS patients. However, it should be remembered that they have various side effects, including they can indirectly affect the function of platelets and blood vessels. More common observed side effects with the drugs are low white blood cell count and slower heart rate. The variety of drugs used in MS is presented in the last meta-analysis, performed by Hamidi et al., including 11 disease-modifying drugs used for treatment of adult patients diagnosed with RRMS, along with their administration form and recommended dose [89].

## Figures and Tables

**Table 1:** List of proven changes in platelet activity and functions in MS

Platelet Investigation in MS and Major Findings	Stage of MS	References
Increased plasma level of β-TG and PF4 (role of platelet degranulation in increasing BBB permeability)	RRMS	[71]
Higher percentage of circulating PMPs (proven pro-inflammatory and prothrombotic properties of PMPs)	RRMS	[46,90]
Elevated level of platelet aggregation (increased platelet hemostatic function)	RRMS	[46]
Raised surface exposure of P-selectin (marker of platelet activation, receptor crucial for cellular interactions)	RRMS	[90]
Increased platelet adhesiveness (changes of platelet hemostatic function)	RRMS	[91]
Augmented plasma level of sP-selectin (marker of permanent activation and consumption of platelets)	RRMS	[92]
Elevated level of PAF in cerebral spinal fluid and plasma (platelet activator and mediator)	RRMS	[93]
Increased expression of P-selectin	SPMS	[43]
Enhanced activation of GPIIb/IIIa (receptor responsible for platelet aggregation)	SPMS	[43]
Higher percentage of circulating PMPs	SPMS	[43]
Augmented formation of platelet aggregates	SPMS	[41,42,43]
Increased platelet adhesiveness	SPMS	[41]
Extensively ROS generation (blood platelets actively participate in oxidative stress existing in SPMS)	SPMS	[41]
Increased cyclooxygenase-dependent arachidonic acid metabolism (the main metabolism pathway in platelets)	SPMS	[42]
High platelet reactivity in response to action of physiological agonists (excessive excitability and sensitivity of platelets)	SPMS	[41,42,43]
